# Dissecting immunophenotypic diversity in multiple myeloma via mass cytometry: a call for harmonized gating strategies

**DOI:** 10.3389/fimmu.2025.1642609

**Published:** 2025-09-04

**Authors:** Mahmoud Singer, Milad Moloudizargari, James Sanchez, Flavia Pichiorri

**Affiliations:** ^1^ Department of Radiological Sciences, School of Medicine, University of California, Irvine, Irvine, CA, United States; ^2^ Judy and Bernard Briskin Center for Multiple Myeloma Research, City of Hope, Duarte, CA, United States; ^3^ Department of Hematologic Malignancies Translational Science, Beckman Research Institute, City of Hope, Duarte, CA, United States

**Keywords:** mass cytometry, flow cytometry, multiple myeloma, immunophenotyping, plasma cell, immune markers, gating, CyTOF

## Abstract

Plasma cell disorders present challenges in phenotypic determination, as they range from monoclonality of plasma cells to multiple myeloma and plasma cell leukemia. According to World Health Organization guidelines, no single aberrant marker is recognized to be uniquely linked to multiple myeloma. The absence of a preset marker panel proven to account for multiple myeloma diversity causes difficulties in diagnosis and clinical research; therefore, the need to create a well-defined panel is urgently needed. For this manuscript, we reviewed the literature on the phenotypic and immunological features that lead to incomplete information and problems in immunophenotyping. We offer proposed solutions for identifying the suitable markers and technology to fill this gap, by using a well-defined gating strategy in a high-dimensional mass cytometry (CyTOF) panel and by next-generation flow cytometry. We analyze pitfalls, starting with sample preparation, selection of the marker panel, gating strategy, cleaning up events, quality control, troubleshooting and validation, and finally, analysis of data. We advance a comprehensive protocol that allows for a detailed analysis of the immunophenotype of myeloma cells. By identifying aberrant markers in the panel, we may be able to facilitate diagnosis and prognosis, ultimately influencing the choice of therapeutic regimens and patients’ overall survival.

## 1 Introduction

Multiple myeloma (MM) is characterized by abnormally high levels of aberrant plasma cells (PCs) in the bone marrow (BM) (1). MM arises from a pre-malignant syndrome known as monoclonal gammopathy of unknown significance (MGUS) (2). Smoldering multiple myeloma (SMM), which is asymptomatic, and ultimately active MM can develop from MGUS over time (3). In a small percentage of patients, MM progresses into plasma cell leukemia (PCL), a condition in which malignant PCs circulate outside of the BM (4).

From a developmental point of view, immature B lymphocytes undergo immunoglobulin heavy and light chain rearrangement before leaving the BM and entering peripheral organs to mature (5). Somatic hypermutation (SHM) and class-switch recombination (CSR) occur during this process, introducing genomic errors (6). Through mutations, SHM improves antigen-antibody affinity, whereas CSR permits the creation of immunoglobulins with various isotypes (7).

Oncogenes are upregulated as a result of translocations, which are frequently found in MM patients and mostly affect the IgH locus (8). The non-hyper-diploid pathway, which is characterized by IgH translocations, and the hyper-diploid system, which involves recurring alterations in chromosomal number, have both been identified as mechanisms in MM oncogenesis (9). It is also common to observe translocations involving the immunoglobulin kappa (IgK) and lambda (IgL) loci, and IgH translocation frequently results in dysregulation of the cyclin D (CCND) genes (10). Additionally, hyper-diploid events occur in 50–60% of MM patients (11). The heterogeneity of MM is also influenced by genetic mutations, clonal evolution, and epigenetic alterations.

With the introduction of immune modulatory drugs (lenalidomide and pomalidomide) (12) and lately with the advent of antibody and T cell-based therapies, such as daratumumab, CAR T cells, and T cell engagers, patients upon relapse exhibit not only further genetic and non-genetic abnormalities in their cancer cells but also immune deregulation. For example, while genetic abnormalities of the targeted antigens have been observed in approximately 30% of the patients treated with T cell engagers (13), this effect is less frequent in patients treated with CAR-T cells, with less than 4% of relapsing patients carrying cancer cells with downregulated or lost surface targeted antigens (14). Nevertheless, most patients currently treated with T cell-based therapy appear to progress due to a dysfunctional immune environment. Patients frequently develop extramedullary disease, in which MM cells maintain the targeted antigen but upon exit from the bone marrow become more resistant to T cell therapies (15, 16). Hence, it is not surprising that, during events associated with both plasma cell degeneration and immune system impairment, researchers observe variations not only in MM cell surface markers, which are often phenotypically different even in the same patient, but also measurable changes in other immune subsets found to be associated with disease progression (17).

For all these reasons, it is becoming more urgent to follow the phenotypical changes of plasma cells and non-cancer immune subsets, using more standardized surface markers and robust and reproducible single-cell acquisition methods, which do not rely on a specific operator gating strategy. It is also important to identify specific populations and how these populations become more heterogeneous during disease progression.

**Figure 1:**
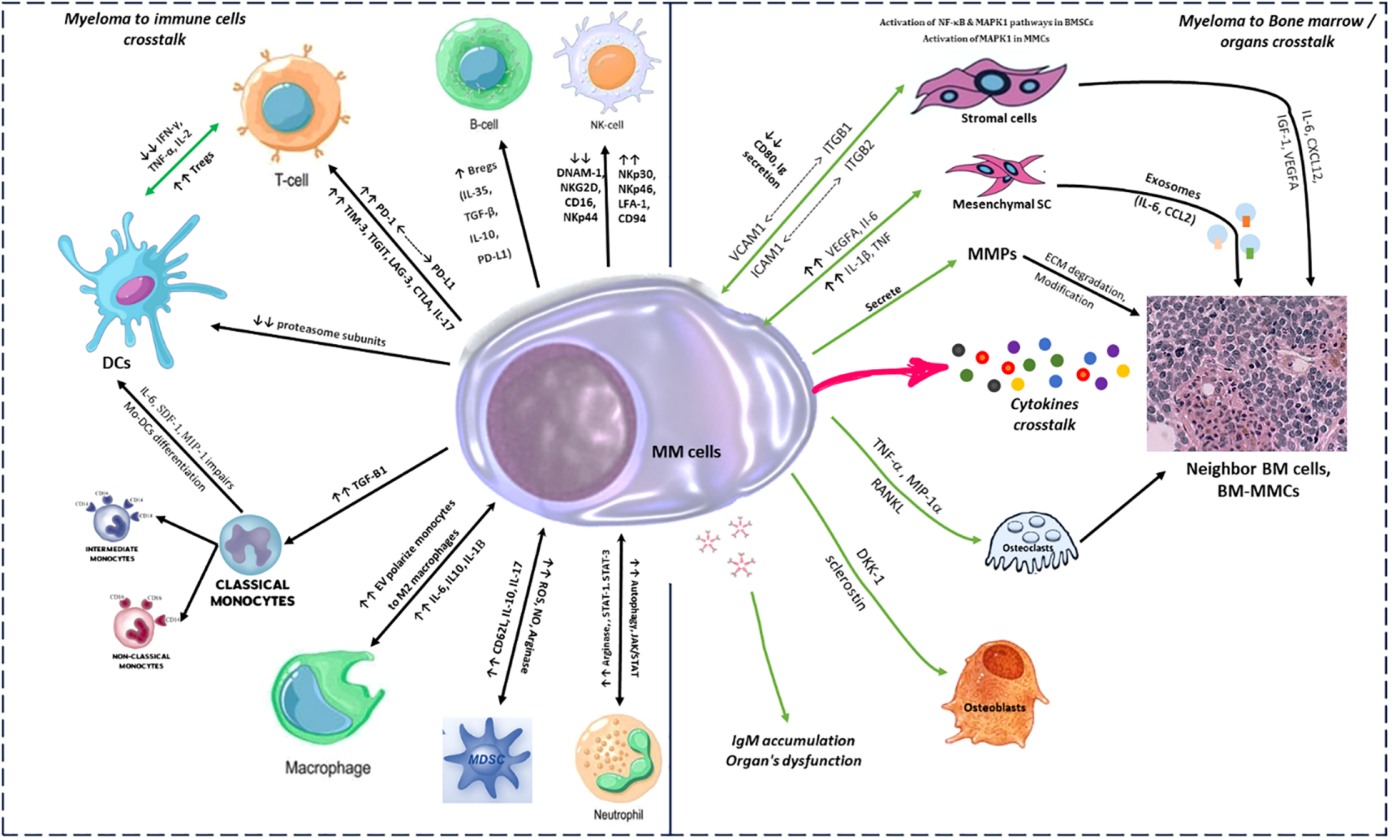
Crosstalk between myeloma cells, immune cells, and bone marrow microenvironment. This figure depicts the intricate crosstalk dynamics between myeloma cells and various components of the immune system, as well as their interaction with key cellular players within the bone marrow microenvironment: Immune cell crosstalk (left panel) and bone marrow organ crosstalk (right panel). NK cells, Natural killer cells; DCs, Dendritic cells; MDSCs, Myeloid-derived suppressor cells; BM-MMCs, Bone marrow multiple myeloma cells.

Although at present the definitive identification of abnormal plasma cells is generally done by flow cytometric analyses, currently there is no universally accepted principle for identifying myeloma cells based on a fixed set of markers. One key factor in this process is the absence of the normally balanced surface expression of immunoglobulin light chains, replaced instead by a monotypic cytoplasmic immunoglobulin light chain. Various markers have been proposed in clinical studies to encompass the plasma cell neoplasms, with each marker believed to play a specific role in the disease. However, it is challenging to fully understand the specific function of each abnormal marker in accurate diagnosis and disease development. For example, it is still unclear how the pattern of expression of CD56, a well-known MM plasma cell surface marker (18), can change with respect to therapeutic resistance and tumor heterogeneity (19). The cross talk between multiple myeloma cancer cells and immune cells in the tumor microenvironment is considered as a key point for correct diagnosis, prognosis and therapeutic monitoring (20, 21). An example of immune cells crosstalk is emphasized in **Figure 1**.

Usually, identifying the immune cells is performed by investigating the immune markers on the targeted immune cells. This process is known as immunophenotyping (IPT) (22). IPT is performed by different techniques, such as flow cytometry or mass cytometry. Both techniques rely on specific bindings of commercially available antibodies to the targeted antigens or markers in order to be investigated qualitatively and quantitatively. The pattern of positive results from the antibody panel identifies the cells and their functions (23). More details about the immunophenotyping techniques will be discussed in detail in Section 3. Some hematological consortia, such as EuroFlow, have contributed to identifying aberrant markers, as well as designing, validating, and optimizing immunological assays for diagnosis, but this approach it is not reliably predictive for disease progression (24).

Lately, the use of single cell RNA sequencing (scRNAseq) platforms, together with the interest of the myeloma community in understanding changes in activated and exhausted T cell phenotypes, have led to some confusion (25), since single cell populations profiled by scRNAseq have not been validated by surface marker expression of corresponding markers. In fact, at present, many studies have not addressed the diverse variations in the immunophenotype of myeloma cells that might contribute to the development of the disease, nor have they validated the presence of specific immune cells throughout different lines of therapies. Therefore, a comprehensive analysis of cells from a large group of patients is required to achieve a more comprehensive and forward-thinking approach to diagnosis.

By incorporating these markers into future diagnostic strategies, we can enrich our understanding of the disease and enable more precise and efficient diagnoses (26–28). Here we review the current literature associated with plasma cell and immune phenotyping in MM, and, based on our laboratory experience, we also provide guidelines for more informative and reproducible immune phenotyping in patients with MM. To our knowledge, we provide for the first time a systematic comparison of the use of flow cytometry versus that of single cell mass cytometry analysis.

## 2 The immune landscape in multiple myeloma and its clinical implications

### 2.1 Immunophenotyping of multiple myeloma

Clinically, the evidence of the malignant clone was and continues to be diagnosed by the presence of M protein, in addition to fluorescent *in situ* hybridization (FISH) studies to detect the potential underlying gene abnormalities (29, 30). Nonetheless, the requirement for tools to monitor the clonal mutation(s) and disease progression at the cellular level revealed the necessity of immunophenotyping techniques. For instance, from a clinical decision-making point of view, IPT has enabled cellular monitoring to detect signs of disease aggravation such as the absence of CD45 expression in MM (28), the overexpression of FOXP3 and CTLA4 in the BM (31), and the overexpression of CD229 on myeloma clonogenic precursors (32). By revealing the patterns of alterations at the cellular level, IPT can provide useful information that could aid in better clinical decision-making.

Additionally, myeloma cells from different patients are heterogeneous, creating unique genotypic patterns (33). The characteristics of each genetic variant affect how the aberrant markers are expressed and consequently determine the phenotype. In clinical hematology, the immunophenotypic and genotypic characterization of peripheral blood and BM-derived myeloma cells is frequently used for initial diagnosis, determination of aberrant expression, and tracking the efficacy of therapeutic regimens (34). Myeloma cells can be recognized using common aberrant markers included in the World Health Organization (WHO) classification of hematological malignancies. However, this list is not all-inclusive. For example, it has been shown that both healthy PCs and malignant myeloma cells exclusively express the plasma cell marker Syndecan-1 (CD138) (35). Recently, it was discovered that Syndecan-1 influences CD4+ T-cell modulation (36). In lymphoid and myeloid lineages, CD38, a transmembrane glycoprotein with ectoenzymatic activity, is typically expressed during different cell maturation and activation processes (37, 38). Additionally, it is expressed on both healthy and cancerous plasma cells (39). PCs are the only population that expresses BCMA (B-cell maturation antigen) (40). The basic phenotypic profile of myeloma cells is distinguished from normal PCs by a CD38-dim CD138-bright expression pattern, together with cytoplasmic light chain kappa or lambda restriction (41). Negative or low expression of CD45, negative CD19 expression in 95% of cases, and negative or low expression of CD27 and CD81 are examples of aberrant expression patterns of MM cells, compared to the phenotype of normal plasma cells (42). Multivariate research has not been able to determine these indicators’ independent prognostic relevance, despite the possibility that some expression patterns are linked to more serious conditions.

Immunophenotyping plays a crucial role in MM, as it provides valuable information about the characteristics of myeloma cells (43). By examining the expression patterns of specific markers, such as CD138, CD38, and BCMA, immunophenotyping helps to accurately identify and differentiate myeloma cells from normal cells. Given the heterogeneity of myeloma cells, it is particularly important to address the need to differentiate them from other plasma cell disorders. Moreover, immunophenotyping enables the monitoring of disease progression and response to therapy (42, 44, 45). Changes in the expression of certain markers can indicate treatment efficacy or disease relapse. It allows clinicians to assess minimal residual disease (MRD), which refers to the presence of small number of residual malignant cells that may not be detectable by conventional diagnostic methods. MRD assessment through immunophenotyping provides valuable insights into treatment outcomes, helps guide therapeutic decisions, and potentially improves patient prognosis. In addition, immunophenotyping contributes to the development of personalized treatment approaches in MM (46). By identifying specific marker expression profiles, clinicians can tailor treatment strategies to target the unique characteristics of each patient’s disease, potentially leading to improved treatment responses and long-term outcomes (47). However, research is still ongoing to establish the prognostic significance of various markers and refine the classification of myeloma cells.

The expression of one marker, CD24, on the surface of clonal PCs is directly correlated with overall survival (48). The expression of another marker, CD45, and its role in disease etiology has also been studied (43). In untreated or relapsed MM or SMM, CD45 expression has been reported to be inversely correlated with disease progression and negatively correlated with high-grade angiogenesis (49). Furthermore, the expression of CD45 on PCs is negatively correlated with their expression of CD138, CD56, and CD54 (49). In terms of marker expression, the expression of two additional markers, MPC-1 and CD49e, was restricted to the mature myeloma cells. Researchers identified immature, intermediate, and mature cells as MPC-1−CD45−/+ CD49e−, MPC-1^+^ CD45^−/+^ CD49e^−^, and MPC-1^+^ CD45^+^ CD49e^+^, respectively (50). Importantly, these morphological and phenotypic classifications do not have established prognostic significance. Nonetheless, understanding the immunophenotypic and genotypic characteristics of myeloma cells is crucial for diagnosing and monitoring the disease.

CD138 (syndecan-1) expression is significantly elevated on the surface of myeloma cells. Elevated CD138+ expression on myeloma cells is associated with enhanced proliferation, prolonged survival, and suppressed apoptosis. This positive correlation is driven by amplified IL-6R signaling, highlighting the role of CD138 in myeloma cell dynamics (51). Nevertheless, low expression of CD138 is associated with tumor fibrosis in bone marrow and is correlated with heparin-binding growth factors that contribute to the pathogenesis of myeloma (52). Blocking CD138 in a myeloma mouse model renders myeloma more vulnerable to bortezomib chemotherapy, resulting in a dramatic decrease in tumor size (51). Combining anti-CD138 antibodies with proteosome inhibitors (bortezomib) or immune modulatory drugs (IMiDs) such as lenalidomide, creates a potentially powerful new strategy to fight myeloma, boosting the immune system’s ability to engulf and destroy both sensitive and resistant cancer cells (53).

An aberrant paraprotein called monoclonal protein is typically produced in patients with MM (54). Both the presence of neoplastic PCs in the BM and the detection of monoclonal protein in the blood or urine are used to establish a diagnosis of MM (54). Although neoplastic myeloma cells constitute the majority of the PCs in patients with MM, some of these patients also have normal or reactive plasma cells. A study by Rawstron et al. revealed that peripheral blood myeloma PCs had much lower levels of CD56 and Syndecan-1 expression compared to levels in bone marrow samples from the same patients (55). These cells were found in 75% (38/51) of patients at presentation, 92% (11/12) of relapsing patients, and 40% (4/10) of stem cell harvests (55). Jeong et al. discovered that a simplified immunophenotyping panel consisting of CD56/CD19/CD138/CD38/CD45 is beneficial in clinical practice for discriminating neoplastic myeloma cells from reactive PCs in a study of 70 individuals with MM (62 newly diagnosed vs. 8 treated) (56). Notably, CD19 expression in neoplastic PCs was negative in both untreated and treated patients, independent of therapy (56). In this regard, it is possible to infer that CD19 is the most helpful marker for distinguishing neoplastic PCs from reactive plasma cells. However, because the immunophenotype of NK cells is frequently identified as CD38+/CD56+/CD19- by flow cytometry, re-gating of cells with dim or negative CD45 expression is required to exclude NK cells. In a study of 132 patients with MM, Robillard et al. discovered that malignant PCs accounted for a median of 97% of total CD38+/CD138+ plasma cells (range: 76.5% to 100%) (1). Their research also found that PCs missing CD19 and expressing either CD56 or CD28 were the most common immunophenotypic population in MM samples (1). Furthermore, Klimienė et al. discovered that a decrease in adhesion molecule expression in MM patients’ BM may contribute to the abundance of circulating PCs in the peripheral blood of patients with relapsed/refractory disease, resulting in the absence of CD49d, CD49e, CD56, CD138, and CD58 markers in circulating peripheral blood plasma samples (57).

**Figure 2:**
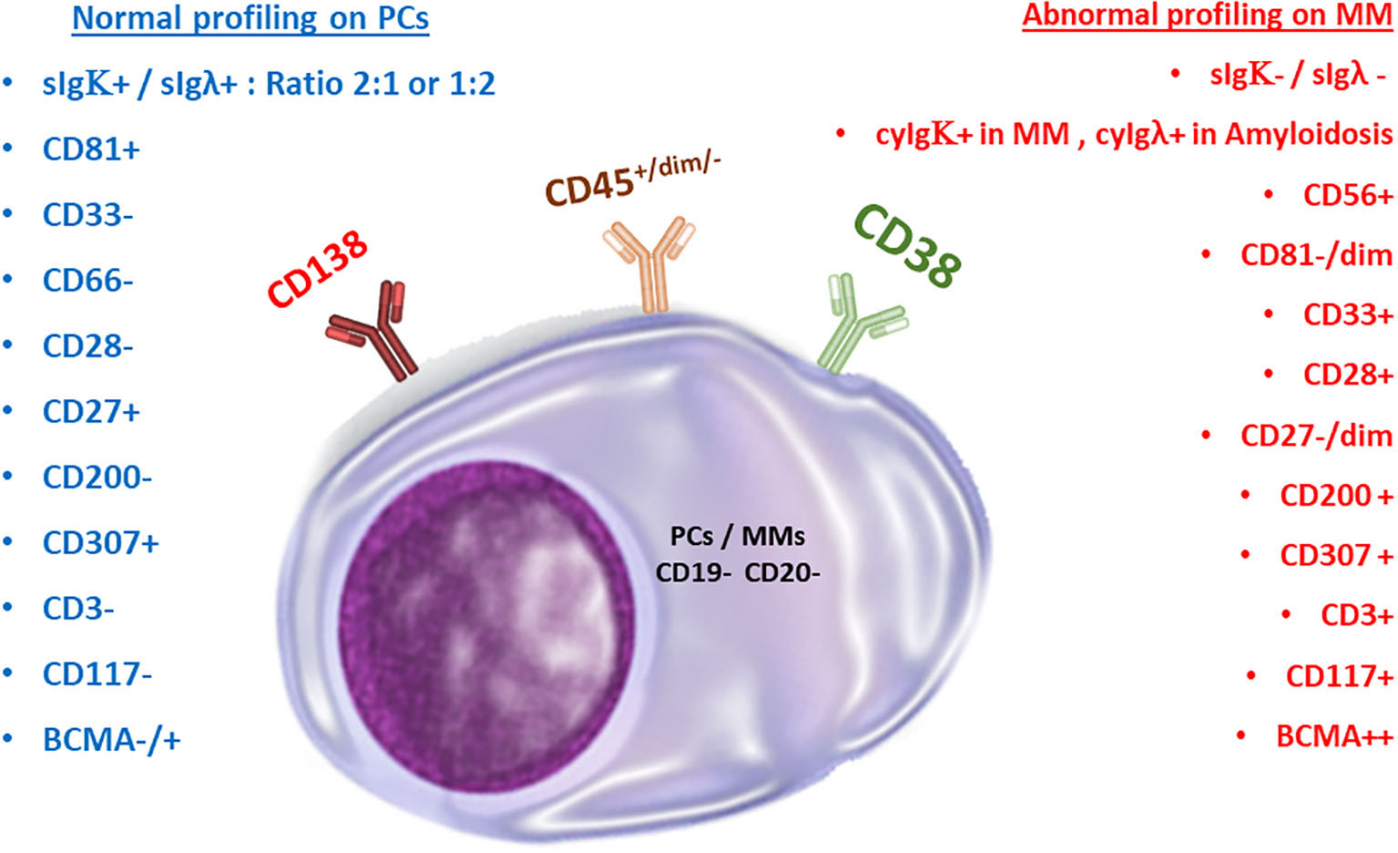
Normal versus malignant plasma cells. Comparison of marker expressions for immunophenotyping purposes.

Several studies correlated the aberrant expression of markers to clinical outcomes: CD117, HLA-DR, and CD33 were found to be independent prognostic factors for decreased progression-free survival (58–61), and the expression of CD200 and CD307 are related to decreased overall survival and poor prognosis, correspondingly (62–65). According to Sanoja-Flores et al., circulating malignant PCs in patients with MM display decreased levels of activation/differentiation-associated antigens such as CD27, Vs38c, and Ki67 (a proliferation marker) (66). Other maturation-associated antigens previously reported to be aberrantly expressed in MM, such as CD19, CD20, and CD45, were found to have no significant differences in the phenotypic profile of peripheral blood versus BM neoplastic plasma cells (66). These findings suggest that antigen expression levels in circulating and BM PCs differ in MM patients, and more research is needed to fully characterize these differences (**Figure 2**).

### 2.2 Major immune populations and gating strategy

**Figure 3:**
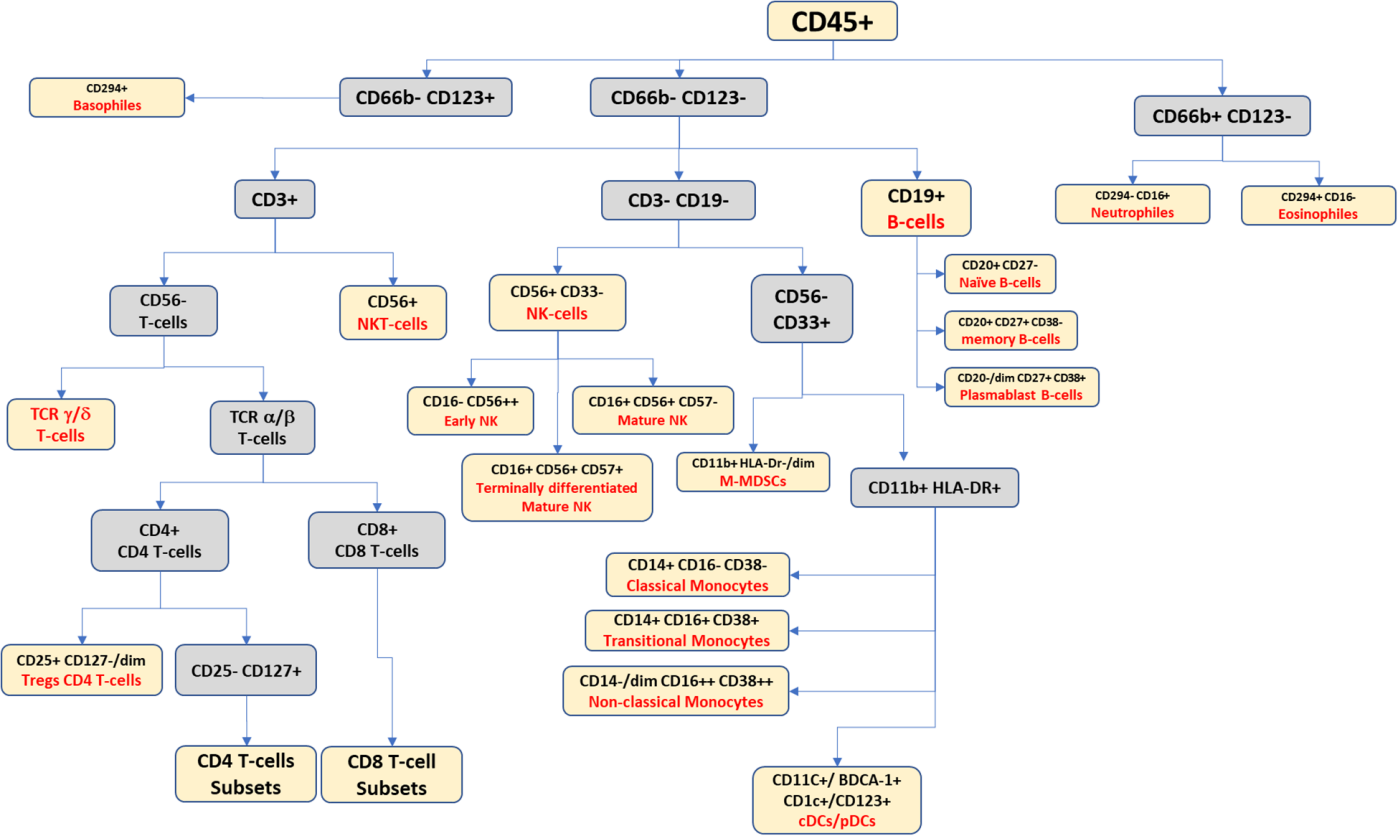
Suggested gating scheme for a high-dimensional panel for identifying the peripheral blood leukocytic continuum.

**Figure 4:**
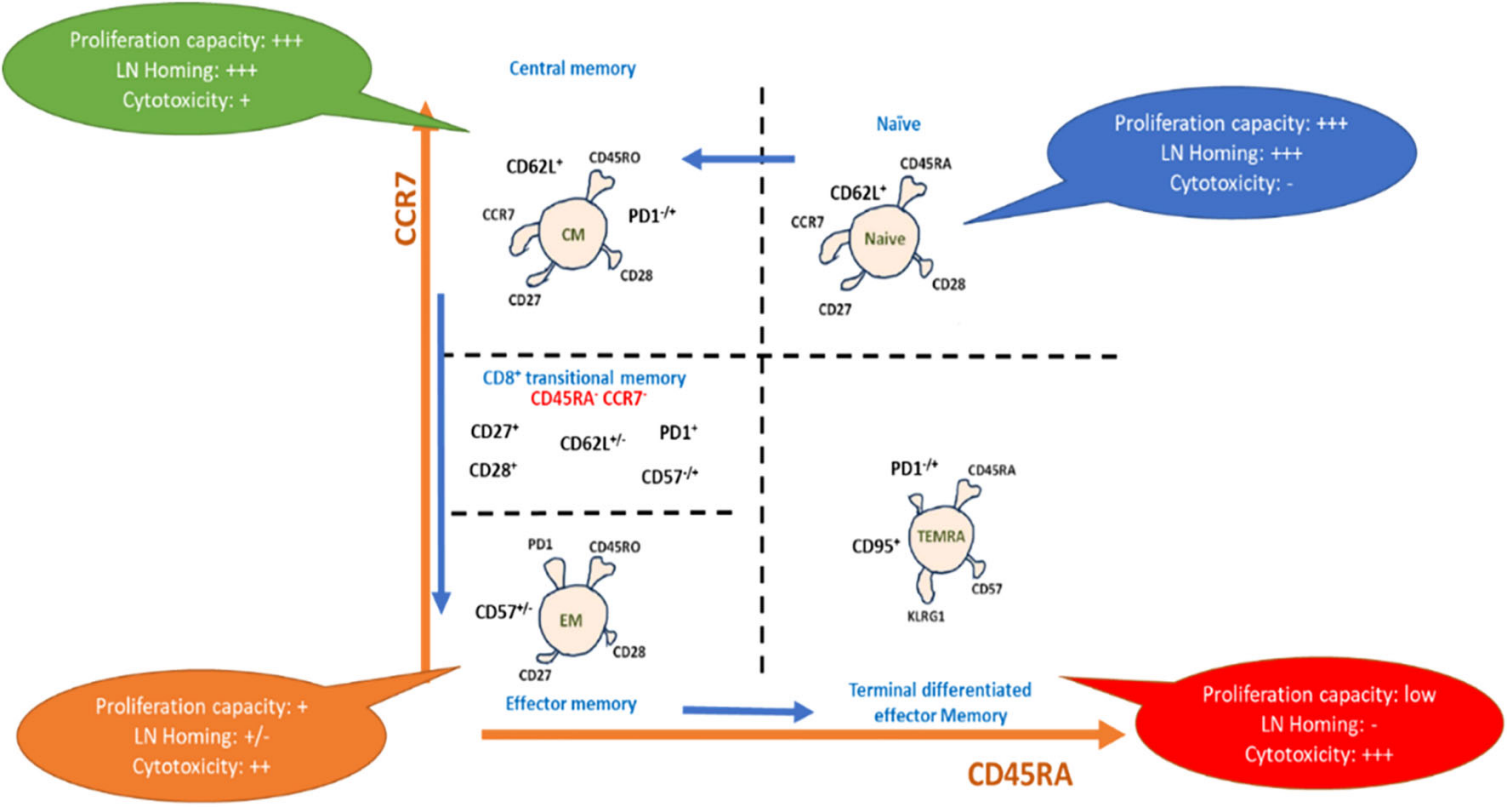
Representative scheme for phenotypic differentiation of T-cells: T-cell maturation after antigen pulsation, showing maturation from naïve T cells to terminal differentiated effector memory T-cells expressing the differential markers with each subset, which drive capacity towards proliferation, homing, and cytotoxicity.

To clarify immune cell subset accumulation and its influence on immune system homeostasis, researchers have employed a range of immunological models, encompassing diverse methodologies and approaches beyond immune cell markers. These models aid in unraveling the complex dynamics and effects of specific immune cell subsets on overall immune system balance. For example, the accumulation of terminally differentiated effector memory T-cells (TEMRA), which are identified as CD3^+^, CD4^+^/CD8^+^, CD127^+/dim^, CD27^-^, CD45RA^+^, and CCR7^-^, may indicate the exhaustion or malfunction of some immune subsets such as NK cells. Another example is related to the difference in the pattern expression of mature NK cells and early NK cells, which are CD56^+^/CD16^+^ and CD56^++^/CD16^-^, respectively. Such findings are important in understanding how immune cells can contribute to tumor progression and for developing new strategies for treating cancer (**Figures 3**, **4**).

Recent research has shown that studying peripheral blood mononuclear cells (PBMCs) in patients with cancer is important for understanding how immune exhaustion mechanisms contribute to tumor progression. Gating PBMCs from patients with MM is similar to the normal gating strategy for healthy donors; however, the detection of circulatory myeloma cells is the main goal in the study of MM patient PBMCs as opposed to regular PBMCs. Herein, we review the marker expression of PBMCs in MM patients. Please refer to **Table 1** for a detailed breakdown of the 43 markers in the CyTOF panel.

**Table 1:** Suggested basic CyTOF panel for the characterization of cancer and immune cells.

Order	Markers	Target cells	Order	Markers	Target cells	Order	Markers	Target cells
1	CD45	Leukocytes	16	CD57	NK/NKT	31	PD-1 (CD279)	Exhaustion markers
2	CD3	T cells	17	CD19	B cells	32	LAG-3	
3	CD4		18	CD20		33	Tim-3	
4	CD8		19	CD27		34	TIGIT	
5	TCR γδ		20	CD66b	Myeloid cells	35	CD138	Plasma cells/Multiple Myeloma (MM)
6	TCR αβ		21	CD294		36	IgGκ	
7	CD25		22	CD123		37	IgGλ	
8	CD127		23	HLA-DR	APCs	38	CD117	
9	CD45RA		24	CD11b	Myeloid cells/Monocytes	39	BCMA	
10	CCR7		25	CD33		40	CD81	
11	CD95		26	CD14	Monocytes	41	CD200	
12	CD28	T cells/MM	27	CD16	Monocytes/NK	42	CD307	
13	TCR Vα24	NKT cells	28	CD11c	DCs/monocytes	43	CD38	MM/B cells/monocytes
14	CD1d		29	Granzyme B	Cytotoxicity markers			
15	CD56	NK/NKT	30	CD107a				

#### 2.2.1 NK cells

In MM PBMCs, in addition to MM cells, a gating approach may also be used to detect additional immune cells in the sample, such as T cells and NK cells (67). These cells are essential for the immunological response to MM and can be utilized to track the patient’s immune health throughout a therapy (68). Numerous studies have been conducted to investigate the marker expressions on NK cells and the presence of NK-related markers in patients with MM. For instance, Carbone et al. discovered that BM-derived myeloma cells from early-stage MM express low levels of the NK cell inhibitory ligand MHC class I and high levels of the NKG2D ligands MHC class I polypeptide-related sequence A (MICA) and MICB, whereas tumors from later-stage disease had the opposite expression pattern and were less susceptible to NK cell cytotoxicity (69). Pazina et al. reported contrasting findings between healthy donor samples and NK cells from patients with MM. They observed decreased expression of DNAM-1, NKG2D, CD16, and NKp44 in MM, whereas increased expression was observed for NKp30, NKp46, and LFA-1 (CD11a) (70). They additionally demonstrated how these variations in marker expression are associated with the stage of MM (70). In this regard, they noted that the changes may be observed in relapsed/refractory MM and post-stem cell transplantation (70). Additionally, they noticed that blood NK cells, particularly in patients with relapsed/refractory disease, expressed more CD69 and SLAMF7 and less CD57 and DNAM-1 (70). Barberi et al. provided additional evidence by demonstrating a significant increase in the CD94dimCD56dim NK cell subpopulation among the total number of NK cells in patients with MM. This subpopulation was found to be notably present in clinical settings associated with MM (71).

#### 2.2.2 Gamma-delta T cells

Under 5% of the peripheral T-cell population are γδ T cells, which are crucial for host defense and tumor monitoring (72). The majority of γδ T lymphocytes are MHC-restricted in their antigen recognition and do not express CD4 or CD8 on their surface (73). These cells also exhibit broad cytolytic activity against tumors and virus-infected cells via the death receptor/ligand (Fas/FasL)-dependent and perforin/granzyme- or granulysin-dependent pathways (74). The activating NK receptor NKG2D and TCR-mediated antigen recognition are both involved in the cytotoxic activity of γδ T lymphocytes (74). These cells play a crucial role in cancer immunosurveillance because they are among the BM infiltrating cells with both innate and adaptive immune cell characteristics (75). There have been few investigations on the ratio or marker expressions of γδ T cells in patients with MM to date; nevertheless, the number of research studies on T cell markers used in the diagnosis of MM patients is growing. According to Sowińska et al., patients with MM have higher percentages of both activated and total CD25-positive γδ-T cells (76). Additionally, they noted that there was a higher percentage of activated CD25+ γδ-T cells (76). In a recent study, Corsale and colleagues explored the role of γδ-T cells in MM, including its early stages (77). Through the development of the first single-cell atlas of γδ T cells in MM, the researchers identified seven distinct γδ T cell clusters. These included two naive subpopulations (CD4-/CD8- and CD4+ γδ T cells), two Granzyme B+ (GZMB) effector/terminally differentiated subpopulations (CD8+/TIGIT+/LAG3+, TIM3+/CD27- and GNLY+/FTH1+ γδ T cells), and two Granzyme K+ (GZMK) memory subpopulations (GZMK+ and C-X-C Motif Chemokine Receptor 3 [CXCR3+] γδ T cells). The study findings revealed a significant decrease in naive γδ T cells (p<0.05), which was coupled with an increase in TIM3 expression, a well-studied exhaustion marker, as MM progressed (77). The researchers also observed a decrease in Vδ2+ T cells compared to levels in healthy donors, as well as an increase in the Vδ1+/Vδ2+ ratio throughout MM progression (77). Additionally, they noticed a positive correlation between the frequency of Vδ2+ T cells and free kappa light chain serum levels in MM.

Interestingly, their research found that both Vδ1+ and Vδ2+ T cell subsets have elevated TIM3 expression, which was linked to altered functioning of both Vδ1+ and Vδ2+ T cells, with decreased TNF-α and IFN-γ production (77). It appears that the TIM3 marker on the surface of T cells may be employed in the marker expression panel for MM diagnosis in the future. Additionally, Brauneck et al. showed that, compared to healthy donors, MM patients’ BM-infiltrating Vδ1+ T cells displayed an increased TEMRA cell population with an aberrant subpopulation of CD27CD45RA++ cells (78). Four markers for these cells—TIGIT, PD-1, TIM-3, and CD39—were expressed by Vδ1 T cells more often than the matching CD4+ T cell population and at levels comparable to those of CD8+ effector cells in hematologic malignancies. When compared to Vδ2 T cells, Vδ1 T cells had a higher frequency of PD-1+, TIGIT+, TIM-3+, and CD39+ cells. These cells were associated with the TEMRA Vδ1 population, which had a significant co-expression of PD-1, TIM-3, and TIGIT (78).

#### 2.2.3 B cells

Clonotypic B lymphocytes (CBLs), also known as progenitors of neoplastic plasma cells, are thoroughly characterized in MM (33). These monoclonal B lymphocytes have identical, rearranged IGH-CDR3 sequences with myeloma cells and have been found in MM patients’ peripheral blood (79). It is agreed that these B cells develop outside of the BM, such as in lymph nodes or other lymphoid organs, and only become PCs after migrating to the BM milieu, which offers a favorable environment for terminal PC development (80). According to Conway et al., circulating B-lineage cells (CBLs), which may serve as the progenitors of cancerous plasma cells, are found in the stem cell harvests from patients with MM. Sixty percent of the patients (12 out of 20) in their study had CBLs that expressed CD34, CD38, CD184, CD31, CD50, and the same immunoglobulin light chain as the patients’ known myeloma cells. The identified CBLs were negative for CD19, CD20, and CD138 (81).

## 3 A practical workflow for high-dimensional cytometry for MM profiling

(Methods available: flow cytometry vs. mass cytometry)

### 3.1 History of mass cytometry

In recent years, different sophisticated single-cell analysis methods that can be used for IPT have evolved. Moldavan is widely regarded as having made the first attempt to count or quantify cells in suspension (1934) (82). Tsutomu Nomizu et al. later found in 1994 that it was feasible to nebulize, dry, and ignite single cells in a hot plasma to form ion clouds detectable by emission spectrometry; this experiment was the first true mass experiment of single cells (83). In 2007, Scott D. Tanner, inspired by flow-cytometry innovations, invented mass cytometry, also known as Cytometry by Time-of-flight (CyTOF), which is the most promising technology for high-dimensional and high-throughput single-cell analysis at the protein level (84). Later, in 2008, Tanner and colleagues depicted that the tandem attachment of a flow cytometer to an inductively coupled plasma mass spectrometry (ICP-MS) machine together with tagging antibodies to metal tags would allow higher multiplexity (85).

### 3.2 Flow cytometry versus mass cytometry

CyTOF may be used to investigate cell phenotypes and function, signaling networks, apoptosis, cell cycle, and a variety of other complicated biological processes. Both CyTOF and flow cytometry (FCM) are founded on the notion of multiplexed single-cell analysis utilizing labeled antibodies. However, there are several key distinctions between the two technologies (**Table 1**). In place of traditional fluorescent labels, CyTOF employs non-biologically accessible metal isotopes with succinct mass spectrometry parameters. Flow cytometry, on the other hand, detects cellular targets using fluorophore-labeled antibodies (86). This change of labeling method allows mass cytometry to examine up to 50 variables simultaneously, avoiding the difficulties associated with overlapping emission spectra, which are common in FCM research (87).

A typical FCM test detects 8–10 distinct markers, while bigger panels are becoming feasible for researchers to develop (88). With the use of next generation flow cytometry (NGF), researchers can now study up to 12 markers in a single staining sample to detect minimal residual disease in MM (70). However, this capacity is less than that of CyTOF, which can identify at least 43 markers in a single sample. In CyTOF staining, the cells are stained by incubating them with a cocktail of probes or antibodies labeled with non-radioactive and non-biological heavy metal isotopes (89). The single-cell suspension is next passed through an argon (Ar) plasma, which atomizes and ionizes the sample, transforming each cell into a cloud containing ions of the elements present within or on the cell surface (90). A high-pass optic extrudes low-mass ions obtained from each cloud, resulting in a cloud containing sole ions linked with the isotope-conjugated probes. The ions are then dispersed by m/z in the time of flight (TOF) chamber before being strengthened and converted into electrical signals (91). Flow cytometry acquisition, by contrast, entails passing intact fluorescently labeled cells through a series of lasers and collecting the emitted light, using specific detectors (92). CyTOF’s exceptional qualities have positioned it as a powerful tool for analyzing considerably diverse clinical samples, while also playing a crucial role in the diagnosis and research of malignancies.

CyTOF detects more markers per sample compared to FCM, whereas FCM has faster acquisition times and preserves cell integrity, allowing them to be recovered for future use (93). Because a CyTOF instrument uses just one detector, no extra tuning or calibration is required for each experiment (94). Moreover, concurrent analysis may be performed from a single tube without the need for single-stained or autofluorescence controls, which is a significant advantage in cases when *in vivo* or clinical research materials are scarce. For example, researchers may profile 30 immune markers from just 300 µL of blood using the Standard BioTools Maxpar^®^ Direct™ Immune Profiling Assay™, a validated, dry-format antibody panel for use on the CyTOF XT™ and Helios™ mass cytometry systems (95). Additionally, mass cytometry data processing does not necessitate post-acquisition data normalization, and although correction of spectrum overlap is essential in flow cytometry, CyTOF requires less compensation due to the use of heavy metal tags rather than fluorochromes. Another advantage of CyTOF is that the metal tags are exceedingly durable and can endure fixation, permeabilization, and freezing without affecting the signal. This feature enables the simultaneous staining of cell surface and intracellular markers, as well as the storage and shipment of labeled samples for multi-site investigations. The use of fluorophores, alternatively, may alter cellular and proteomic changes in the cells, which may influence the immunophenotypic signature as well as the intracellular and functional targets.

On the other hand, one present disadvantage of CyTOF is that it is a relatively new technique when compared to FCM. Heavy metal-conjugated antibodies are less frequently commercially accessible than fluorophore-conjugated antibodies (96). As a result, if a large mass cytometry panel is sought, a major amount of a researcher’s effort may be allocated to self-reagent production, such as in-house antibody conjugation. Furthermore, the need to sort the subpopulations discovered by high-dimensional phenotyping for further downstream research, such as single-cell RNAseq or functional studies, is widespread and addressable with full-spectrum FCM rather than CyTOF (97). Flow rates in CyTOF (500 events/s) are substantially slower than in fluidic or acoustic flow cytometers (3,000 and 35,000, respectively) (98). Also, the Helios™ CyTOF cytometers are fairly expensive (98). When this cost is combined with the additional expense of conjugating antibodies to heavy metal tags, many researchers cannot accommodate the expenditures.

However, CyTOF is beneficial in clinical scenarios, where a large number of variables on a small sample size may be examined (99). Indeed, CyTOF analysis has been used in several clinical research studies throughout the world to examine various areas of human illness to better understand and enhance the effectiveness of clinical treatments. The most significant barrier that remains is inherent in the complexity of the data processing, which necessitates extensive biostatistical and bioinformatic abilities, which frequently complicates its implementation in a clinical situation. **Table 2** summarizes the advantages and disadvantages of FCM and CyTOF (99).

**Table 2:** Pros and cons of next generation flow cytometry, spectral unmixing flow cytometry and CyTOF for immunophenotyping.

	Next generation flow cytometry	Spectral unmixing flow cytometry	CyTOF (Mass cytometry by time of flight)	References
Identification tag	Fluorochromes	Fluorochromes	Rare heavy metal isotopes (only 15 metals)	(100, 101)
Specificity Level	Highly specific	Highly specific	Highly specific	(102)
Basis of Sensitivity	quantum-efficient fluorochromes	separating overlapping spectral signals (Coarse Wavelength Division Multiplexing [CWDM] semiconductor detector arrays)	Lanthanide metals and their isotopes	(103, 104)
Acquisition	Up to 20,000 events/second	Up to 20,000 events/second	Up to 300 events/second	(102, 105)
Multiplicity	Generally, 12 markers (in development to reach up to 35 channels)	Up to 40 colors in combination, with 64 fluorescence detectors	Up to 43 markers (in development to reach up to 150 channels)	(99, 106, 107)
Standardization	CE, FDA approved	China and Europe	N/A	(108)
Background noise	Auto-fluorescence of cells	Auto-fluorescence of cells	Very low background, comes from oxides and metal impurities	(109)
Discrimination of population based on positivity	Moderate	Moderate	Higher	(99, 110, 111)
Channel resolution	Wide range	Wide range	Wider range	(112)
Compensation	Mandatory	Auto Compensation by beads	No compensation required	(110, 113, 114)
Percentage of overlap between fluorochromes	0 – 50%	≤ 98%	<2%	(108, 115, 116)
Validity for samples acquiring after staining	Few hours due to photobleaching process	Few hours due to photobleaching process	Up to 2 weeks without loss of signal	(117–119)
Cryopreservation of stained samples	Not stable	Not stable	Stable for up to 1 month	(120, 121)
Disadvantages/Limitations	- Limited with fluorochrome spillovers	- Computationally complex; still fluorescence - Limited when using dyes and antibody markers	- Absence of physical property of cells (cell size and granularity) - High loss of cells during acquisition - No cell sorting - Expensive	(122–125)

**Figure 5:**
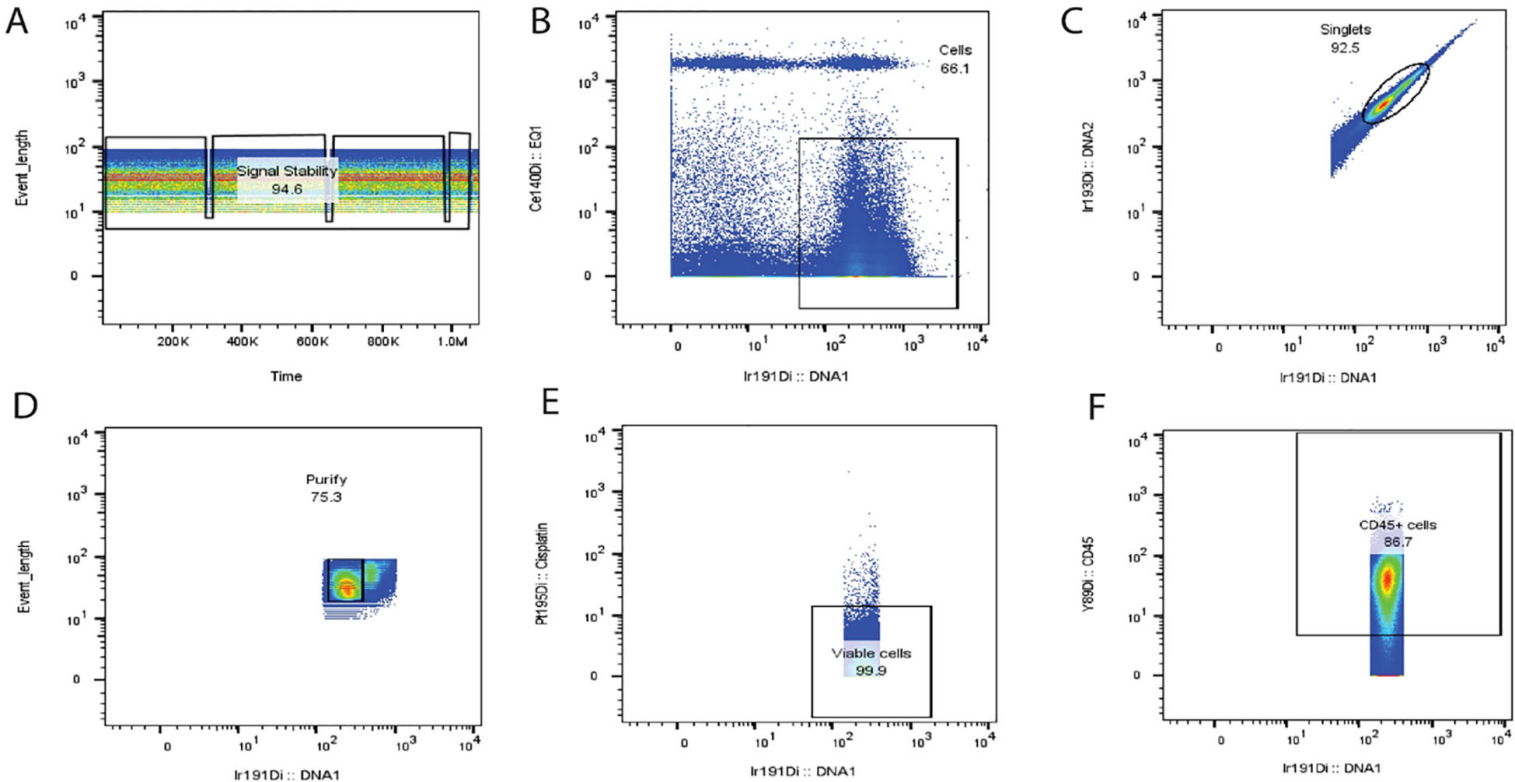
Suggested steps for cleaning up CyTOF data. Based on our extensive experience in mitigating non-specific signals arising from unstable data, beads, cell doublets, non-viable cells, and other sources of unknown noise, we have implemented a robust data-cleaning protocol. Following data normalization, the cleaning process involves a series of gating steps to isolate the desired signal events [as depicted in **(A)**]. Firstly, we identify and gate the stable signal events **(A)**, followed by the selection of positive intercalator signals, specifically Ir191 and Rh103, while excluding the negative bead population Ce140 **(B)**. Subsequently, the selected events from the prior gates are further refined by singlet population selection **(C, D)**. In unique cases where frozen samples are used, monocytes may exhibit aggregation and appear within the doublet region. In such instances, it is better to include these doublets in the analysis and conduct cleaning at each sequential gating step. The final stage of the cleaning process involves the exclusion of non-viable cells **(E)**, ensuring that only viable and relevant data are retained for subsequent analysis. Ultimately, the gating of backbone markers, typically CD45, is initiated for the final analysis **(F)**. PeacoQC analysis for removing diverse anomalies associated with data acquisition across various platforms also effectively identifies and filters out low-quality events within stable density peaks. This comprehensive approach to data cleaning and signal refinement is essential for ensuring the accuracy and reliability of downstream analyses and result interpretation. Sources for FCS files used for illustration were imported from the flow repositories (127–129).

Based on our experience with and the guidelines for use of flow cytometry (126) for cleaning up the non-specific signals generated from unstable signals, beads, cell doublets, non-viable cells, and other unknown noises, we adopted these methods for cleaning up the fcs data files of CyTOF samples: After data normalization, the next clean-up step is to gate on the stable signal events (**Figure 5A**), followed by a selection of positive intercalator signal (Ir191/Rh103), while excluding the negative beads population (Ce140) (**Figure 5B**). The outcome events of the previously selected gates may be further refined by a selection of singlets population (**Figures 5C, D**). In exceptional cases when using frozen samples, monocytes are shown to be aggregated and appear in the doublet’s region; therefore, it is better to include these doublets and subsequently perform cleaning at each step of sequential gating. The last step is the exclusion of non-viable cells (**Figure 5E**) before starting the gating of backbone markers (usually CD45) for analysis (**Figure 5F**).

## 4 Challenges and future directions

### 4.1 Challenges and pitfalls in the immunophenotyping of myeloma

The need for the use of next-generation flow cytometry and CyTOF has increased recently in immunophenotyping research, notably in the analysis of larger panels of markers (130). The use of larger panels enables a more thorough characterization of immune subsets and offers a deep comprehension of the intricate interactions between various cell types under both healthy and diseased circumstances (122).

CD3, a crucial T cell marker, is found to be expressed abnormally on neoplastic plasma cells (131). This overlap between different markers, either normally or abnormally expressed, makes comprehensive immunophenotypic profiling and functional analysis very challenging and requires that all plasma cell subpopulations be considered. In addition to these considerations, alternate markers or gating techniques can also be investigated. Unusual and less frequent cases, such as MM that is CD138-negative, might render typical flow cytometry procedures difficult (132). Additionally, the use of certain monoclonal antibodies, such as daratumumab (a CD38-targeted monoclonal antibody), during therapy may affect plasma cells’ CD38 positivity (133). For a precise diagnosis and evaluation of the effectiveness of the treatment, such variations require careful observation and reporting. To minimize diagnostic errors, pathologists should also be attentive to aberrant expression patterns and ensure they address any discrepancies or imbalances in their interpretations. Aberrant expression patterns can be recognized and addressed with quality control procedures and staying current with new research discoveries.

Heterogeneous marker distribution in myeloma can also result in missed diagnoses, particularly when inadequate sample size or sampling errors are involved. To reduce the likelihood of misdiagnoses, it is crucial to consider the total morphological picture and combine other diagnostic methods, including imaging or genetic analysis. Due to the intricate modifications needed for color correction in flow cytometry, simultaneous multi-color immunophenotyping might be difficult. Data quality can be affected, and variability can be introduced by the operator’s subjective manipulation of instrument controls. In clinical flow cytometry laboratories, using automated technology for color adjustment during data gathering can lower operator-dependent variability and raise uniformity. Making the right choices for color compensation can be aided by using software such as Bagwell and Watson’s automated technique.

### 4.2 Common pitfalls and challenges in using CyTOF

#### 4.2.1 Sample quality importance for correct immunophenotyping

Sample preparation is also significant, since it is essential to collect reliable and accurate immunophenotypic data. Throughout the experimental workflow, proper sample processing enables the maintenance of cell viability, reduces artifacts, and maintains the integrity of cellular markers. The use of processed stale blood samples poses a technical risk that has a substantial impact on the results. Non-fresh samples may exhibit altered phenotypes due to metabolic changes that may affect cell activity and/or exhaustion (134). Therefore, it is recommended to prepare and isolate leukocytes from fresh samples before any immunophenotyping attempt. Additionally, when freshly taken samples are unavailable, the samples need to have been properly thawed and frozen. Maintaining cell viability requires proper freezing and thawing techniques. The release of free DNA and RNA from non-viable cells and cell debris can result in cell death and aggregation (135). Sample integrity can be preserved by using DNase, heparin as an anticoagulant, or a washing step using Benzonase Nuclease. Based on our experience, for the best outcomes, a concentration of up to 50 units of Benzonase per ml of full RPMI-1640 medium can be used. The effectiveness and reliability of immunophenotypic analyses have been improved by this powerful DNA and RNA degrading enzyme, which has shown superior performance in avoiding cell aggregation and maintaining the integrity of surface markers (136, 137).

#### 4.2.2 Unified staining protocol for reproducibility

Additionally, it is important to use an established staining procedure with accurate antibody titration and proper timing for each incubation step. Accurate and reproducible results can be achieved by adhering to the manufacturer’s recommendations and avoiding superfluous vortexing. To acquire high-quality data, it is crucial to validate and optimize the Helios machine’s acquisition settings, including quality control validation, barium background, and rationalized sample acquisition speed. Between 250 and 600 events/second can be gathered by increasing the number of pushes and adjusting the acquisition speed to improve separation (138). Ultimately, data analysis and comparability can be enhanced by normalizing all the FCS files from the same experiment at the same time.

#### 4.2.3 Quality control importance in IPT to avoid false results

It is indeed promising that a novel multi-omics approach has been meticulously developed to elucidate the surfaceome dynamics observed in MM cells, thereby offering valuable insights into potential immunotherapeutic strategies (139). However, it is important to acknowledge that, when comparing patients with MM to cell line models, the inherent heterogeneity in the surfaceome poses a significant challenge in the straightforward translation of these findings into effective therapeutic models. In recent years, there has been a significant expansion in the development of comprehensive antibody panels, comprising, for example, over 40 antibodies employed in CyTOF analyses. Nevertheless, the presence of signal streak outliers has posed a challenge to the precision of the results obtained. To address this issue, researchers have utilized several algorithms, such as Flowclean and FlowAI, to rectify these signal streak aberrations, primarily focusing on flow cytometry data (140). However, these algorithms exhibit limitations in their applicability, as they are tailored exclusively for flow cytometric data. An updated and versatile data cleaning tool, PeacoQC, has emerged as a solution to address these limitations. PeacoQC not only excels in removing diverse anomalies associated with data acquisition across various platforms, but also effectively identifies and filters out low-quality events within stable density peaks. This capability is achieved through the innovative utilization of an isolation tree and Median Absolute Deviation (MAD) distances (141, 142). PeacoQC is distinguished by its adaptability across a range of cytometric technologies, including flow cytometry, mass cytometry, and spectral cytometry datasets. One of its characteristic features is the ability to evaluate and discriminate flow rate from signal stability independently, providing a comprehensive approach to enhancing data quality (142). In conclusion, there are several challenges and risks associated with the immunophenotyping of myeloma, which can be overcome by taking into account the aforementioned factors and the use of cutting-edge technologies, optimizing sample processing, and validating staining protocols.

### 4.3 Future directions in using novel algorithms in analysis to accommodate the level of multiplexity

To ensure the quality and reliability of the data acquired, researchers are faced with new hurdles when handling larger antibody panels in CyTOF. The lack of analytical tools that can successfully accommodate these larger panels is one of the main issues (143). The number of parameters that a conventional flow cytometry analysis software can manage is usually limited, and the computing power required to process the data is another limiting factor (144). It is crucial to have new processes to contend with the challenges of larger panels. To address the increased complexity of the data produced by more extensive antibody panels, the field requires the development of advanced algorithms and data processing methods (145). Improvements to the physical infrastructure, such as increasing processing and storage capacity, are among the other requirements for an efficient analysis of the data associated with large antibody panels (146). Furthermore, it is essential to recruit reliable validation strategies in addition to new analytical instruments. This approach entails ensuring that the antibody staining is accurate and reliable, optimizing the panel design to reduce spectral overlap and maximizing the resolution, and setting the right quality control methods in place along the experimental workflow.

Spectral unmixing flow cytometry offers advantages in studying the immune map of multiple myeloma to characterize the immunohematology complexities. Using 40 markers will help to provide a deep comprehensive view of the hematopoietic and immune cell landscapes at the single-cell level, such as plasma cell populations, changes in immune cell profiling, and their microenvironment. This multiplexing in addition to different unbiased analysis algorithms may quantify rare cell types, discern differences in cell abundance and phenotype across patients, and find any subtle changes in the immune landscape that lead to either disease progression or treatment response (147). With over 40 markers, diagnostic panels are critical to MM management. These panels help distinguish MM from other plasma cell disorders, identify high-risk MGUS and smoldering MM, and are essential for post-treatment MRD assessment (42). Both spectral unmixing flow cytometry or CyTOF will help to capture detailed immunophenotypic profiles that can also contribute to the definition of antigenic profiles that impact prognosis, as well as the identification of new therapeutic targets, potentially leading to more personalized treatment approaches (42).

The precise quantitative and qualitative data required to analyze marker expression kinetics, population similarity, and sample comparisons at the minor population level can only be obtained through the implementation of several crucial algorithms. The cells can be visualized in a 3D projection alongside the overall stained markers and then represented in a 2D figure in an unbiased manner (147). Using machine learning algorithms and this tSNE programming helps to improve the tSNE plotting. As a result of several developments, optimized tSNE (Opt-NSE) emerges as an automated solution for analysis to improve the visualization of large sets of data (148, 149). Based on tSNE, vast comparative algorithms are created with regard to events clustering (such as FlowSOM, SPADE) and statistical characterization (such as CITRUS) (150–152).

## 5 Conclusion

Determining the phenotype of plasma cell disorders is often complex, and its correlation with disease prognosis is not adequately established. The need to create a well-descriptive panel to identify a comprehensive aberrant pattern may be useful in monitoring the patient’s immune response as well as tracking cancer cell mutational status. CyTOF can offer higher dimensional panels with comparatively more analytical tools to delve into the immune continuum of patients with MM. Updating our analytical tools may advance the choice of therapeutic regimens and extend patients’ overall survival.
